# Change in urgent psychiatric consultations during the first lockdown in Venezia: a multicenter, retrospective study

**DOI:** 10.1192/j.eurpsy.2023.1255

**Published:** 2023-07-19

**Authors:** S. Rosson, A. Sanseverino, F. Baggio, G. Zanuttigh, J. Tubini, M. Bianco, M. De Rossi, E. Gallo

**Affiliations:** 1 Department of Mental Health, Azienda ULSS3 Serenissima, VENEZIA; 2 Department of Cardiac, Thoracic, Vascular Sciences and Public Health, University of Padova, Padova, Italy

## Abstract

**Introduction:**

The COVID-19 pandemic has affected the mental health of the global population (Dragioti *et al.* J Med Virol 2022;94(5):1935-49). The first lockdown brought the hardest and most sudden impact on work, educational, social, and recreational activities. Moreover, the fruition of mental health services was restricted, and non-urgent appointments were delayed or converted into telepsychiatry. Thus, it was reasonable to hypothesize different trends of urgent consultations regarding mental health.

**Objectives:**

To detect quantitative and qualitative changes in patients presenting to our Emergency Departments (ED) during the early phase of the pandemic compared to the previous year.

**Methods:**

We conducted a retrospective, multicenter study in Venezia (historical center, mainland) through systematically reviewing the psychiatric consultations in our ED, during the first 16 weeks since 8-Mar-2020 and the same period of 2019. The protocol was approved by the local Ethics Committee as UPSI-19 (Urgent PSychiatric consultations In COVID-19). The statistical analysis was conducted with the software R; Interval Risk Ratio (IRR) with 95% CI was calculated for absolute frequency, primary diagnosis, leading symptoms, and outcomes of these consultations.

**Results:**

In the early phase of the pandemic, in our ED we assisted to a significant decrease in psychiatric consultations: 372 vs 441, IRR=0.84(0.73-0.96). Data revealed a reduction of referral for suicidal behavior (IRR=0.52(0.33-0.80)) and anxiety symptoms (IRR=0.60(0.42-0.87)). Primary diagnoses of patients were not different between the two periods explored. There was a slight increase in admissions (150 vs 121), and a significant decrease in less severe clinical pictures.

**Conclusions:**

In the timeframe considered, we assisted to a significant decrease in referrals from the ED, possibly related both to fewer non-locals and to less frequent non-severe presentations. Despite the type of patients (for underlying diagnoses) remained unmodified, an interesting reduction of anxiety symptoms and suicidal behavior was noticed. Literature from ED studies during the first wave are consistent with our finding regarding the number of visits; suicide attempts seemed unmodified or decreased elsewhere (Giner *et al*. Curr Psychiatry Rep 2022;24(1):1-10). Limitations of our study include peculiarities of the Venetian territory, limited sample and time of observation. Future directions encompass the integration with data from the community setting and later developments.

**Disclosure of Interest:**

None Declared

